# Content in Simple Signalling Systems

**DOI:** 10.1093/bjps/axw036

**Published:** 2017-06-22

**Authors:** Nicholas Shea, Peter Godfrey-Smith, Rosa Cao

**Affiliations:** 1 Institute of Philosophy, School of Advanced Study University of London London, UK; 2 Graduate Center City University of New York New York, USA and Unit for the History and Philosophy of Science University of Sydney Sydney, Australia; 3 Philosophy Department Stanford University Standford, USA

## Abstract

Our understanding of communication and its evolution has advanced significantly through the study of simple models involving interacting senders and receivers of signals. Many theorists have thought that the resources of mathematical information theory are all that are needed to capture the meaning or content that is being communicated in these systems. However, the way theorists routinely talk about the models implicitly draws on a conception of content that is richer than bare informational content, especially in contexts where false content is important. This article shows that this concept can be made precise by defining a notion of functional content that captures the degree to which different states of the world are involved in stabilizing senders’ and receivers’ use of a signal at equilibrium. A series of case studies is used to contrast functional content with informational content, and to illustrate the explanatory role and limitations of this definition of functional content.

**1** *Introduction*
**2** *Modelling Framework*
**3** *Two Kinds of Content*
    **3.1** *Informational content*
    **3.2** *Functional content*
**4** *Cases*
    **4.1** *Case 1: Simplest case*
    **4.2** *Case 2: Partial pooling*
    **4.3** *Case 3: Bottleneck*
    **4.4** *Case 4: Partial common interest*
    **4.5** *Case 5: Deception*
    **4.6** *Case 6: A further problem arising from divergent interests*
**5** *Discussion*
*Appendix*

## 1 Introduction

Recent years have seen dramatic advances in our understanding of communication and its evolution, through new models developed in biology, philosophy, linguistics, and economics. The models in these areas take different forms, but many can be seen as having a common theme. They show how sign-using interactions between senders and receivers are stabilized by means of selection processes that bear on sender and receiver behaviours.^1^

Communication is usually thought to involve the production of signs or representations that have meaning, or content of some kind. Writers working in, or influenced by, the mathematical theory of information have sometimes wanted to set these issues aside, as irrelevant or positively unhelpful. Freeman Dyson claims that information theory’s central dogma is that ‘meaning is irrelevant’ (Dyson [2011]; see also Shannon [1948], p. 379). Another recent discussion concurs:

When information theorists think about coding, they are not thinking about semantic properties. All of the semantic properties are stuffed into the codebook, the interface between source structure and channel structure, which to information theorists is as interesting as a phonebook is to sociologists. (Bergstrom and Rosvall [2011], p. 171)

In an important treatment of this topic, Skyrms ([2010]) argues that although questions of meaning and content are worth considering, a straightforward extension of basic ideas in information theory suffices to handle them. Signals have informational content when they change the probabilities of states of the world, or of a receiver's actions. Informational content exists whenever probabilities are changed in this way, regardless of what role the messages play; the informational content of a signal is represented by a vector that records, for each possible world state, how much the signal changes the probability of that state compared to its antecedent probability. This, for Skyrms, is all we need to recognize when thinking about content.

We agree that one way of understanding the content of signals in sender–receiver systems is by applying information-theoretic ideas in this way. But, we argue, there is also another approach to the interpretation of signals in systems of this kind, one tied to the way that actions guided by a signal have consequences that can stabilize signing behaviours.

Note first that whether signals have informational content, in Skyrms's sense, does not depend on whether they are part of a system with signs being used successfully to coordinate action with the state of the world. They would still carry informational content even if they were part of a system in which the use of signals was not achieving anything useful at all, the system was far from equilibrium, and signals were giving rise to behaviours poorly matched with the world. Existing discussions in the modelling literature sometimes acknowledge, explicitly or tacitly, the appeal of a notion of content that is tied to the maintenance of equilibria in some way.

One response to this situation is to look for a view of content that combines informational and ‘functional’ considerations of this kind. This may well be fruitful, but our approach in this article is different. We will treat ‘informational content’ and ‘functional content’ as two separate and useful concepts, with distinct explanatory roles. Informational content involves probabilistic associations between signs and the world; functional content involves relations between signs and the world that figure in the stabilization of a system of sign use. The aim of the article is to analyse content in a way that is not guided by common-sense intuitions but by consideration of which notions of content are useful when thinking about signalling systems and their evolution.

The next section outlines the modelling framework used in the article. Subsequent sections describe the two kinds of content and then proceed through a series of cases that illustrate the two kinds of content and their complementary roles. The article aims to motivate a distinction between informational and functional content, but does not purport to be the last word on how functional content should best be formalized. In the discussion of some cases, we acknowledge some problems for our proposed formalization and provisionally sketch some ways it could be amended to overcome those limitations.

## 2 Modelling Framework

Our discussion is concerned with signalling systems that have the structure of a Lewis signalling game. David Lewis ([1969]) gave a model of signalling in which we assume two agents, a sender and a receiver, where the sender has access to information about the state of the world, but cannot act on it except to send signals of some kind. The receiver can see only the signals, but can act in a way that generates payoffs for both sides. The payoffs resulting from a receiver's pairing of an act with a state of the world might be the same for sender and receiver or they might differ.

Lewis assumed that sender and receiver policies were rationally chosen in a situation of common knowledge. Brian Skyrms ([1996], [2010]) gave an evolutionary recasting of Lewis's model. Rational choice was replaced by natural selection, or in some cases by simple forms of learning. Evolution, learning, and choice are all processes in which the consequences of behaviours can ‘feed back’ and re-shape the rules governing behaviour at later time-steps. The sender modifies (or maintains) its sender's rule, which maps states of the world to signals; the receiver modifies (or maintains) its receiver's rule, which maps signals to acts. When a combination of a sender's and a receiver's rule is such that neither side can change their rule unilaterally and be better off, given what the other is doing, the system is in a Nash equilibrium. When a combination of rules is such that any unilateral change makes the changer worse off, the system is in a strict Nash equilibrium.

The Lewis–Skyrms model is related to models discussed in economics (Crawford and Sobel [1982]; Farrell and Rabin [1996]) and in evolutionary biology (Maynard Smith and Harper [1995], [2003]; Bergstrom and Lachmann [1998]; Zollman *et al.* [2013]). Models in economics have explored issues like honesty in advertising and the use of signals to help maintain cooperation (Spence [1973]; Robson [1990]). Honesty in signalling has also been a focus of biological and evolutionary models, investigating especially the way that a cost associated with a signal can enforce honesty. Both evolutionary and economic modelling have explored the consequences of divergence of interests between senders and receivers for the possibility and nature of signalling. Our discussion will be focused on the set-up described by Lewis and Skyrms, but many of our conclusions can be extended more broadly.

Formally, we are concerned with situations where there is an exogenously determined state of the world, {*S*_1_, *S*_2_, …}, a sender who can detect this state and has a range of signals or messages available, {*M*_1_, *M*_2_, …}, and a receiver who can see the signals and may use them when choosing among available actions, {*A*_1,_*A*_2_, …}. States of the world are associated with objective probabilities, P(*S_i_*). Combinations of acts and states are associated with payoffs for each agent, represented by matrices (introduced below in Section 4). A sender's rule is a mapping from states to messages; a receiver's rule is a mapping from messages to acts. Both these rules may be ‘pure’ or ‘mixed’; a sender may, for example, respond to *S*_1_ by always producing *M*_1_ (a pure strategy), or perhaps by producing *M*_1_ with probability *p* and *M*_2_ with probability 1 − *p* (a mixed strategy). Our analysis of cases in this article will be simple. In general, we will note combinations of senders' and receivers' rules that are equilibrium states, states where neither side has any incentive to change their behaviour. In some cases, drawing on the work of others, we will give a richer description, which notes how a case behaves under some rule of evolutionary change. Much of our discussion is intended to be neutral, though, about the details of the selection process shaping the sender's and receiver's behaviours.

## 3 Two Kinds of Content

### 3.1 Informational content

An appealing way to think about the content of signals in sender–receiver systems is to draw on concepts from information theory (Shannon [1948]; Dretske [1981]). Signals carry information about states of the world when they change the probabilities of those states (Skyrms [2010]). The term ‘change’ here should not be understood as involving strange causal relations between signal and state, but merely the fact that the probability of a state conditional upon the signal is different from the unconditional probability of that state. A signal has content when it tells us something about how the world is, where ‘tells’ is a matter of changing probabilities, providing evidence. Dretske ([1981]) developed a view of this kind, but for a signal to have content, he required that it raise the probability of some state of the world to one. A signal says that the world is in *S*_2_, for example, if the probability of the world being in *S*_2_, given the signal, is one, and its probability independent of the signal is less than one. Skyrms ([2010]) outlines a more general view of the informational content of signals. A signal has informational content if it changes the probabilities of at least some states of the world, and its content is given by all the changes it makes to the probabilities of those states. So if a signal raises the probability of *S*_2_, but does not bring it to one, it can still tell us something about *S*_2_. For Skyrms, the kind of content where some states’ probabilities are reduced to zero is a special case (which he labels ‘propositional content’).

Skyrms adopts a particular format for representing the changes made by a signal to the probabilities of a set of states. For a set of states, {*S_i_*}, the content of a signal, *M_j_*, is constituted by the changes made to the probability of each state by the signal, where each ‘change’ is measured as the binary logarithm of the ratio of the conditional to the unconditional probability of that state. That is, the content of *M_j_* is:

$$<\;{\text{log}}_{2}(P(S_{1}|M_{j})/P(S_{1})),{\;\;\text{log}\;}_{2}(P(S_{2}|M_{j})/P(S_{2})),\ldots {\;\;\text{log}\;}_{2}(P(S_{i}|M_{j})/P(S_{i})),\ldots >.$$

So the content of a message is a vector. In the special case where a message reduces the probabilities of some states to zero, Skyrms labels those states in the vector with minus infinity. For example, if a message eliminates all but one of four initially equiprobable states, the content will be of the form <−∞, 2, −∞, −∞>. Then the content can be given in a familiar propositional form by disjoining the remaining states. Here the content of the signal is *S*_2_; in another case, it might be *S*_2_*-or-S*_3_, and so on.

We follow Skyrms in thinking of content in general as given by a vector, with contents that definitively rule out some states being a special case, but we will do this with a simpler method than Skyrms's. For us, the informational content of message *M* is the vector of post-signal probabilities of the states, P(*S_i_*|*M*). So in the case given above, where a message eliminates all but one of four initially equiprobable states, the content will be of the form <0, 1, 0, 0>. Both Skyrms's and our method have advantages and disadvantages (Godfrey-Smith [2012]). A disadvantage with using post-signal probabilities to represent content is the fact that the content vector is well defined even if the message has not changed any probabilities, so P(*S_i_*) = P(*S_i_*|*M*) for all *i*. Our response is to stipulate that in cases where all the states have their probabilities unchanged by a signal, the signal has no informational content. Our use of the posterior probability vector is motivated in part by the way it makes possible some formal comparisons between informational and functional content.

So the informational content of a signal is the distribution of probabilities of states of the world, conditional on that signal, with the proviso that at least some of these probabilities differ from the unconditional probabilities of the states. The informational properties of signals depend solely, then, on the unconditional probabilities of the states, together with the sender's rule. In cases where a message rules out some states of the world, a narrative summary of the content can be given (in the form ‘*S*_1_’, or ‘*S*_1_*–or–S*_2_’). When no states of the world are ruled out, a narrative summary would be vacuous. As Skyrms notes, a signal can carry information about both the states of the world perceived by the sender and about acts produced by the receiver. Here we will only discuss informational content about the state of the world.

### 3.2 Functional content

Signals have informational content (in both Skyrms's and our sense) whether a sender–receiver system is at equilibrium or not, and whether the signals are doing anything useful for the users or not. If a sender and receiver have rules configured so that the sender maps states to signals one-to-one, and the receiver maps signals to acts one-to-one, but in a way that guarantees that the act produced is the worst one possible in each state, signals have the same informational content they would have if the sender was performing the same mapping of states to signals, but the receiver was producing the best act in each state. Informational content is insensitive to facts about how well things are going and whether the system is at any kind of equilibrium.

This is not in any sense a problem for the notion of informational content. However, many writers have formed the view that content, of at least some variety, is dependent on those further factors. This might be seen as recognition of a richer concept of ‘meaning’ than mere informational content. For example, Simon Huttegger takes linguistic meaning (‘the linguistic component of the truth of a statement’) to be fixed by the conventions of meaning (Huttegger [2007a], p. 2), which are strict Nash equilibria of signalling games (p. 9).^2^ Similarly, William Harms identifies ‘primitive content’ with pairs of dispositions of senders to produce signals and receivers to act on signals, when such pairs have been stabilized by evolution or learning (Harms [2004]).^3^

These thoughts suggest that there is an additional way of thinking about content in signalling systems, having to do with the stabilization of the setup and the beneficial consequences of sender–receiver coordination. In the biological literature on animal signalling, the concept of ‘functional reference’ has been applied to such situations (Macedonia and Evans [1993]; Scarantino [2013]; cf. Wheeler and Fischer [2012]). In philosophy, both information-theoretic relationships and relationships involving success and stabilization of representation-using systems have been employed as the basis for general theories of content. They are usually seen as rivals: informational theories analyse content in terms of correlations between representations and states (Dretske [1981]; Fodor [1990]); teleosemantic theories hold that the content of a representation derives from the way a ‘consumer’ system acts on the representation to produce adaptive behaviour that has been relevant to the stabilization of that representation-using system (Millikan [1984], [1989]; Papineau [1984], [1993]). For example, when a vervet monkey sees a snake and makes a particular sound, ‘consumer’ monkeys run for cover in the trees. This has been useful in cases where the sound was produced in the presence of snakes, so ‘Snake!' is the content of the sound, even if those cases are rare and many sounds are false alarms. Some philosophical theories of content rely on both functional and informational properties in combination (Neander [forthcoming]; Price [2001]; Shea [2007]).^4^

Those earlier debates about informational and teleofunctional theories were not generally carried out in the context of a sender–receiver model of the kind we are concerned with here.^5^ Rather than aiming for a choice between informational and functional properties, or a ‘gluing together’ of them, here we look at the idea that there are two kinds of content that messages can have in a sender–receiver system. One kind is derived from informational properties of the message—the way messages correlate with states of the world—and the other arises from the role the message plays in stabilization of the system through some process of selection.

Accordingly, we define functional content as follows: The messages in a sender–receiver system have functional content only if the system is at an equilibrium maintained by some selection process.^6^ If it is, then for each signal *M*, we ask whether there is a behaviour (or distribution over behaviours) of the receiver specific to *M*, in the sense that the receiver responds differently to *M* than it does to some other available signal. (Note that this allows that the receiver may respond the same way to some other signal *M*’, but rules out that the receiver should respond the same way to all signals in the system.) If so, we look at whether there is a specific state of the world that obtains on some occasions when the message is sent, where the relation between that state of the world and the behaviours produced by the message contributes to the stabilization of those sender and receiver behaviours. If so, that state is the content of *M*. If the receiver’s behaviour in response to *M* is stabilized by the obtaining of more than one world state on different occasions, the signal will have a disjunctive content involving all those world states.

In the case of informational content, we followed Skyrms in saying that content in general is given by a vector. We apply the same principle to functional content. The informational content vector takes the form of a list of entries that sum to one—the posterior probabilities of states of the world. The functional content vector we use here is also a list of entries that sum to one, though these entries are not probabilities. Whereas the informational content vector for a signal gives, for each state, how probable it is in the light of the signal, the functional content vector gives, for each state, the degree of involvement of that state in the stabilization of the sender's and receiver's behaviours regarding that signal. In the simplest cases, as with the vervet's ‘Snake!' alarm call, there is just one state of the world whose obtaining figures in the stabilization of the system. But suppose that this particular alarm call has been mostly useful when there have been snakes around, and has afforded some protection when there are wild dogs around instead. Then the call has some functional involvement with both states.

More precisely, we define the functional content vector for a message in relation to baseline payoffs for the sender and receiver obtained in the absence of signalling. (The following recipe is expressed more formally in the appendix.) The baseline for each agent is the agent’s average payoff in a situation where the receiver adopts the best strategy available to it without conditioning its behaviour on any signals (cf. Scott-Phillips *et al.* [2012], p. 1944). Non-zero entries in the vector for the functional content of a message correspond to states in which the message is sent and both agents receive above-baseline payoffs, given the receiver's rule for that message. For each such state, we calculate the difference between the sender payoffs received in that state and its baseline; we calculate the corresponding difference for the receiver. When necessary, we take the smaller difference to yield a single value for each state. (See below for discussion of when this minimum must be considered and what role it plays.) These values are weighted by the posterior probabilities of the states, given the signal, and normalized to sum to one.^7^ The result is a vector representing the relative importance of each state to the stabilization of the sender and receiver rules for that message. This can be seen as a measure of the degree of involvement of the message with each state, given how the message is produced and used to guide action. (Some complications arise when sender and receiver payoffs differ, but do not differ so much that only one payoff is above baseline—we discuss these below.)

In that first presentation, we assumed that the receiver performs a single action in response to *M*. A receiver might ‘mix’ its behavioural responses to *M*, however, producing (say) act *A*_1_ half the time and *A*_2_ the rest of the time. In those cases, each action is analysed separately in the way outlined above, and the results are averaged, weighted by the probability that the receiver will produce the action in response to *M*.

The two kinds of content have the same form—distributions over states of the world, one reflecting posterior probabilities and one reflecting functional involvement. In cases where one or more entries are zero, a narrative summary of the content is available. This applies to both kinds of content. For example, a vector of the form <0, 0.6, 0.4> can be summarized as *S*_2_*-or-S*_3_. Vectors with no non-zero entries do not have a non-vacuous narrative summary.

Sometimes the informational content and functional content will coincide and, in some cases, will diverge. Lastly, the truth—the state of the world on an occasion when a signal is produced—can also be represented in the same form as the two kinds of content, with a distribution summing (trivially) to one. If, for example, there are three possible states of the world, *S*_1_, *S*_2_, and *S*_3_, and on some occasion *S*_2_ is the actual state, this can be represented in a vector: <0, 1, 0>. So the state of the world, the informational content of a signal, and the functional content of a signal all have the same form.

Before showing how these definitions play out in some cases from the existing literature, we will comment briefly on two alternative proposals. Harms ([2010]) illustrates a rather different way of connecting Lewis-style signalling games with philosophical work on teleosemantic theories of content. Harms does not use vectors to capture functional content. Our treatment also differs from Harms's in making functional content partly a matter of the relative magnitude of the payoffs received in different states. Harms has a different focus, driven by concerns about how the world can be divided into states objectively. As a result, he dispenses with states of the world external to the sender–receiver system and characterizes his model only by reference to states of the sender’s sensory apparatus and the payoffs that are received in those states. Functional contents are regions of a state space defined by the range of available sensory states and payoffs. There is not scope here to explore the extent to which Harms's approach is a rival to the one we develop here and the extent to which they are complementary.

Our functional content vector is broadly in the spirit of Oliver Lean's ([2014]) ‘informational functions’. However, Lean casts his approach as contrasting with teleosemantic accounts of semantic information in biology, arguing that greater clarity is achieved by analysing function separately from information, and treating information in the style of Shannon. By contrast, we argue that a function-related notion of content is a useful resource for analysing communication in signalling systems.

We now turn to examples that illustrate the different roles of the two kinds of content.

## 4 Cases

### 4.1 Case 1: The simplest case

**Figure 1.**
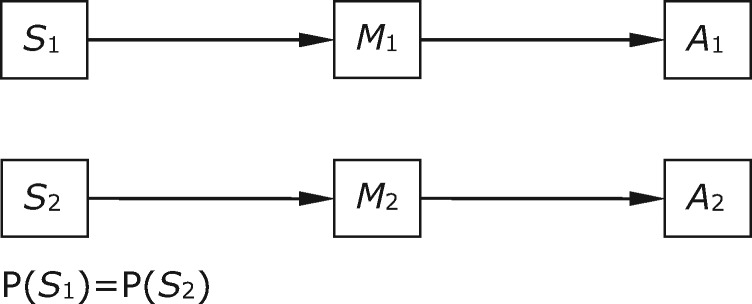
A signalling system in the case where there are two world states, two acts, and two signals available.

The simplest case is where there are two world states, two signals, and two acts, and the world states are equally probable. Both agents receive a positive payoff when *A*_1_ is produced in *S*_1_ and the same when *A*_2_ is produced in *S*_2_, and neither receives a payoff otherwise. There are four possible sender strategies and four possible receiver strategies (leaving aside mixed strategies). Two of these are combinations of sender and receiver behaviours in which maximum payoff is achieved by both parties on every trial because signals are used to perfectly correlate the receiver's actions with the state of the world. One of these signalling systems is shown in Figure 1; here the sender invariably sends *M*_1_ in response to *S*_1_, the receiver produces *A*_1_ in response, and so on. The other simply swaps *M*_1_ with *M*_2_ in Figure 1. These are the only strict Nash equilibria of the game. Recent models have also shown that evolutionary processes can guide populations of various kinds to these equilibrium states (Huttegger *et al.* [2010]; Skyrms [2010]).

At the equilibrium shown in Figure 1, signal *M*_1_ makes state *S*_1_ certain and completely rules out state *S*_2_, so the post-signal probabilities are <1,0>. The functional content of *M*_1_ is determined, as explained above, by examining the behaviour of the receiver specific to that signal and noting which pairing of messages to states contributes to the stabilization of the system. In this case, the functional contents of both messages are the same as their informational contents; *M*_1_ is produced always and only in *S*_1_, and *M*_1_ gives rise to *A*_1_, which contributes to the stabilization of the system if and only if *S*_1_ obtains. So the contents are as set out in Table 1.

**Table 1.** Relations between informational and functional content for Case 1

		Informational content	Functional content
Messages	*M*_1_	<1, 0>; *S*_1_	<1, 0>; *S*_1_
	*M*_2_	<0, 1>; *S*_2_	<0, 1>; *S*_2_

Contents are given first in vector form and then in a narrative summary.

### 4.2 Case 2: Partial pooling

Even in simple situations like the set-up above, as soon as the probabilities of the two world states differ, informative signalling may become evolutionarily unlikely. In a pooling equilibrium, the sender sends the same signal in both states and so the signal is completely uninformative about the state of the world. Correspondingly, the receiver ignores the signal and performs the same action regardless. These equilibria exist even when the probabilities of states are equal, but they are more evolutionarily relevant when those probabilities are unequal, because in evolutionary models of situations in which the probabilities are unequal, populations do frequently end up in pooling equilibria. These are models in which each agent in the population plays the sender role half the time and the receiver role half the time, receiving payoffs according to the matching of receiver actions with states, and the population evolves by the replicator dynamics (Huttegger *et al.* [2010]; Skyrms [2010]). Pooling is a common outcome because agents implementing pairs of behaviours that constitute a signalling system incur a cost when they encounter pooling agents, since they then condition their behaviour on a completely uninformative signal. Simply performing the behaviour best suited to the most probable state is sufficiently profitable that it may be hard for signalling to invade.

Suppose we have a case like this, with *S*_1_ much more probable than *S*_2_, where the sender sends *M*_1_ in every state and the receiver performs *A*_1_, regardless of what they see. Then the signals do not change the probabilities of states of the world at all, in which case no signal has informational content in our sense. As the receiver performs the same acts in response to all messages, no signal has functional content either. As described above, a signal only has functional content when there is a characteristic behaviour resulting from that signal that plays a role in the stabilization of the system. Here, no signals are associated with characteristic behaviours in this sense.

**Figure 2.**
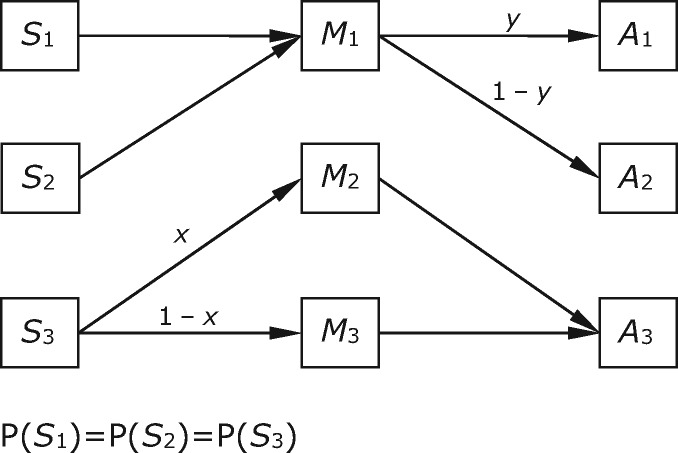
A case of partial pooling in a system with three states, signals, and acts.

Once there are three states, signals, and acts, partial pooling becomes possible, where the sender pools two world states together under the same signal, but sends a different signal in the third state.^8^ In the strategies shown in Figure 2, a case drawn from (Skyrms [2010]), the sender sends *M*_1_ in response to both *S*_1_ and *S*_2_, and mixes *M*_2_ and *M*_3_ in response to *S*_3_, with probabilities *x* and 1 − *x*, respectively. The receiver maps both *M*_2_ and *M*_3_ to act *A*_3_, and mixes its response to *M*_1_, producing *A*_1_ and *A*_2_ with probabilities *y* and 1 − *y*, respectively.^9^ Here we assume again that the three states of the world are equally probable. The assumptions about payoffs are as they were above: both actors receive a payoff in world state *S_i_* if and only if act *A_i_* is produced, with the magnitude of the payoffs the same in each case. In evolutionary simulations of the kind described above, some populations of senders and receivers do end up at equilibria of this kind (Huttegger *et al.* [2010]; Skyrms [2010]).

In this case, message *M*_1_ shifts the probabilities equally towards both *S*_1_ and *S*_2_, and *M*_2_ and *M*_3_ both shift the probabilities towards *S*_3_, giving rise to the informational contents set out in Table 2.

**Table 2.** Relations between informational and functional content for Case 2

		Informational content	Functional content
Messages	*M*_1_	<0.5, 0.5, 0>; *S*_1_*-or-S*_2_	if *y* ≥ ^2^/_3_,
			<1, 0, 0>; *S*_1_
			if ^2^/_3_ > *y* > ^1^/_3_,
			<3*y* − 1, 2 − 3*y*, 0>; *S*_1_*-or-S*_2_
			if *y* ≤ ^1^/_3_,
			<0, 1, 0>; *S*_2_
	*M*_2_	<0, 0, 1>; *S*_3_	<0, 0, 1>; *S*_3_
	*M*_3_	<0, 0, 1>; *S*_3_	<0, 0, 1>; *S*_3_

Contents are given first in vector form and then in a narrative summary.

The receiver’s behaviour in response to *M*_1_ is to perform a mixture of *A*_1_ and *A*_2_. What is the functional content of *M*_1_? What is the condition whose obtaining on occasions where *M*_1_ is acted on explains the success of this mixed policy of behaviour? The answer is that this depends on the value of *y*. In some situations, both *S*_1_ and *S*_2_ are involved in generating payoffs that are above baseline, given the mix of actions performed in response to *M*_1_. In those cases, the condition is disjunctive: the functional content of *M*_1_ is *S*_1_*-or-S*_2_. This is a rough narrative summary, though. The functional content vector for *M*_1_ is more specific, as it reflects the fact that proportion 3*y *− 1 of the payoffs received at equilibrium are in world state *S*_1_ and 2 − 3*y* in *S*_2_.

In other situations, when *y* is close to an extreme value, one or other of *S*_1_ and *S*_2_ does not play such a role, and the functional content is not disjunctive. The contents of the three signals are set out in Table 2.

As shown in Table 2, this case features divergence between functional and informational content, where the degree of divergence depends on *y*. When expressed in narrative terms, the functional content is stronger, for high and low values of *y*.

### 4.3 Case 3: Bottleneck

**Figure 3.**
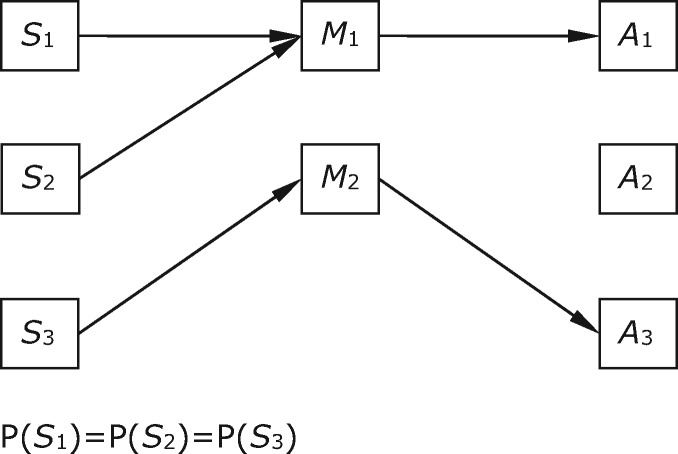
Sender and receiver behaviours in a ‘bottleneck’ case, with fewer messages than states.

We now consider a different situation in which sender and receiver payoffs are suboptimal but the system can be at equilibrium. This is a case where there are not enough messages available to cover all the states—there are three world states but only two signals available by which to communicate about them. In the solution in Figure 3, action *A*_2_ is never performed, and in *S*_2_, the agents receive the suboptimal reward of four, obtainable by performing *A*_1_ in *S*_2_. This combination of behaviours produces the best outcome possible in the situation (Skyrms [2010], p. 113).

In this and subsequent cases, the details of payoffs are important. We represent them in a table with entries for the payoff received for each action in each world state. In Table 3, sender and receiver payoffs do not differ from one another.

**Table 3.** Payoffs for a ‘bottleneck’ case

			Acts	
		*A*_1_	*A*_2_	*A*_3_
	***S***_1_	7	0	2
States	***S***_2_	4	6	0
	***S***_3_	0	5	10

The strategy in Figure 3 is structurally similar to the strategy in the previous case (Figure 2) in that it pools two world states. This is another case in which the functional content of *M*_1_ differs from its informational content. Though the behaviour produced in response to *M*_1_ does yield some payoff in *S*_2_, this payoff does not exceed the baseline achievable in the absence of signalling. For *M*_2_, in contrast, the functional and informational contents line up entirely (Table 4).

**Table 4.** Relations between informational and functional content for Case 3

		Informational content	Functional content
Messages	*M*_1_	<0.5, 0.5, 0>; *S*_1_*-or-S*_2_	<1, 0, 0>; *S*_1_
	*M*_2_	<0, 0, 1>; *S*_3_	<0, 0, 1>; *S*_3_

Contents are given first in vector form and then in a narrative summary.

### 4.4 Case 4: Partial common interest

**Figure 4.**
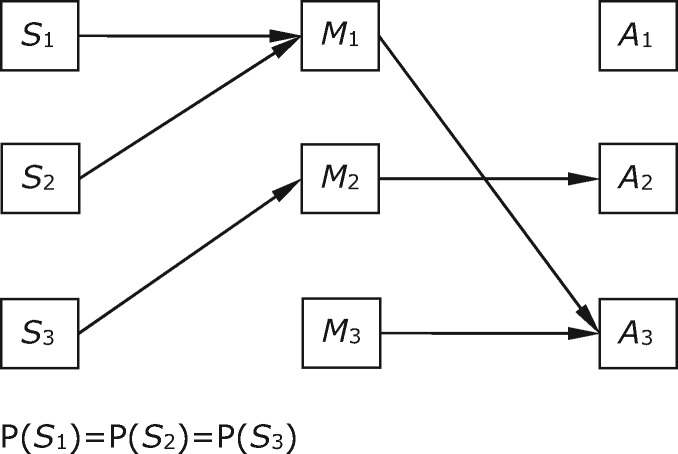
A case of partial common interest.

We now consider a game in which the payoffs for sender and receiver differ such that their interests are not fully aligned. They agree about the best action in one of the states (*S*_3_), but in the other two, they have a different preference order.^10^ The payoffs are shown in Table 5.^11^ In Figure 4, a combination of sender and receiver rules is shown that yields an equilibrium for this system (Skyrms [2010], p. 80). The sender uses *M*_1_ to rule out *S*_3_ and raise the probability of *S*_1_ and *S*_2_ equally, inducing the receiver to perform the sender’s preferred action in both states, since that action also pays off reasonably well for the receiver. The sender has an incentive not to differentiate *S*_1_ and *S*_2_ because then the receiver would perform its preferred action for each, to the detriment of the sender. So here imperfect alignment of interests produces partial pooling of states by the sender.

**Table 5.** Payoffs in a case of partial common interest

			Acts	
		*A*_1_	*A*_2_	*A*_3_
	***S***_1_	2, 10	0, 0	10, 8
States	***S***_2_	0, 0	2, 10	10, 8
	***S***_3_	0, 0	10, 10	0, 0

Payoffs in each cell are to sender and receiver, respectively.

One reason researchers have been interested in cases where the interests of senders and receiver differ is because it raises the possibility of deception. However deception might be analysed in detail, at least the paradigm cases involve the sender using signals to achieve payoffs that run counter to the best interests of the receiver by inducing the receiver to perform actions that are not well aligned, given their interests, with the state of the world. Skyrms argues that the equilibrium shown in Figure 4 is a case of deception. We do not agree. What is true in this case is that signal *M*_1_ carries less than perfect information about the actual state, failing to distinguish *S*_1_ from *S*_2_. The receiver produces a cover-all behaviour that generates reasonably good payoffs in both *S*_1_ and *S*_2_. In no circumstance does the receiver produce an action well suited only to one state when a different state obtains. The receiver's payoffs are always above their baseline. The functional and informational contents of the two messages used are given in Table 6. The sender is conveying and the receiver is acting on a true disjunctive content every time *M*_1_ is sent (*S*_1_*-or-S*_2_).

**Table 6.** Relations between informational and functional content for Case 4

		Informational content	Functional content
Messages	*M*_1_	<0.5, 0.5, 0>; *S*_1_*-or-S*_2_	<0.5, 0.5, 0>; *S*_1_*-or-S*_2_
	*M*_2_	<0, 0, 1>; *S*_3_	<0, 0, 1>; *S*_3_

Contents are given first in vector form and then in a narrative summary.

Part of the reason Skyrms holds that this is a case of deception is the fact that when *M*_1_ is sent, there is misinformation; the probability of a non-actual state of the world is raised by the signal (Skyrms [2010], p. 80). However, that was also true in the two cases of pooling discussed above (Cases 2 and 3), where signals, again, did not discriminate all states. We think this case (Case 4), is merely a case of strategic withholding of information by the sender, a phenomenon quite distinct from deception. We will next consider a case that we do regard as one of *bona fide* deception.

### 4.5 Case 5: Deception

To illustrate the possibility of genuine deception, we consider a signalling game relevant to animal communication, modifying a game discussed in (Zollman *et al.* [2013], Figures 2 and 3, Table 2). Suppose senders are males and receivers are females, and males signal to advertise their quality. Males can be high or low quality. Males always prefer to mate, whereas females prefer to mate only with high-quality males. (These contexts involving display are assumed to not be the only contexts in which females can mate; uniform refusal to mate by a female in these contexts does not imply zero fitness.) Suppose too that males have a signal available that is more costly for low-quality than high-quality individuals to send. (The payoffs are represented in Table 7.) Then a stable signalling system can evolve in which males reliably signal their quality and females condition their mating behaviour on the signal.

**Table 7.** Payoffs in the deception case described in Section 4.5

		Acts	
		*A*_1_ (mate)	*A*_2_ (not mate)
States	***S*_1_** (high-quality male)	2, 2	1, 1
	***S*_2_** (low-quality male)	2, 0	1, 1

Payoffs in each cell are to sender (male) and receiver (female), respectively.

**Figure 5.**
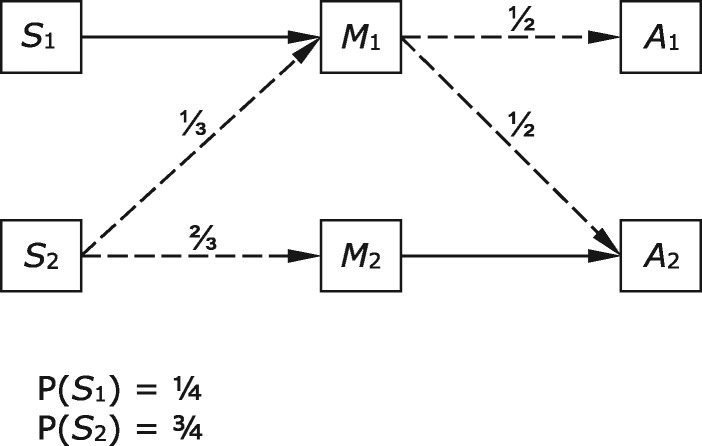
A case of deception: a hybrid equilibrium of the case described in Section 4.5. *S*_1_ and *S*_2_ are the possible states of the male sender. *M*_1_ is a costly signal. It costs ½ for low-quality males (*S*_2_) to send *M*_1_, but only ¼ for high-quality males (*S*_1_). Signal *M*_2_ has no cost.

There is also a ‘hybrid equilibrium’ of this game, which we will focus on here, in which both senders and receivers sometimes mix their behaviours and sometimes do not. High-quality male senders always send the more costly high-quality signal. Low-quality males randomize, sending the high-cost signal in some cases and the low-cost low-quality signal on other occasions. On the receiver side, males who send the low-quality signal are always rejected and those who send the high-quality signal are accepted with some probability and rejected the rest of the time. Whether a hybrid equilibrium exists depends on the parameter values—payoffs, costs of signals, and the frequency of high-quality males—and this equilibrium will involve a specific mix of sender and receiver behaviours. One example of a set of parameters for which an equilibrium exists is given in Table 7 and Figure 5. Here, the low-quality males send the high-cost signal with probability ⅓ and high-cost signals are accepted with probability ½. This combination of sender and receiver strategies is a Nash equilibrium.^12^

The informational and functional contents of messages at this hybrid equilibrium are set out in Table 8. Message *M*_1_ has no propositional informational content, because no state is ruled out by the message. However, it does have a functional content that is propositional: *S*_1_. This is the only state in which the receiver's rule at equilibrium generates for both sides an above-baseline payoff. As a consequence, when *M*_1_ is sent in *S*_2_, which does happen some of the time, this message has false propositional content. It says the world is in *S*_1_ when in fact the world is in *S*_2_. False propositional content is quite different from what Skyrms called ‘misinformation’. The case in Figure 5 is the only case so far in which a signal sometimes has false propositional content, while misinformation in Skyrms's sense is found also in cases with bottlenecks and pooling (Sections 4.2–4.4).

**Table 8.** Relations between informational and functional content for Case 5

		Informational content	Functional content
Messages	*M*_1_	<0.5, 0.5>; no propositional content	<1, 0>; *S*_1_
	*M*_2_	<0, 1>; *S*_2_	None

Contents are given first in vector form and then where possible in a narrative summary.

We understand deception to occur when a message with a false content is sent and the receiver is induced to behave in a way that benefits the sender and harms the receiver. ‘Deception’ in this sense is a success term; it can be distinguished from attempted deception, which occurs when a message with false content is sent in a way that has the potential to benefit the sender at the expense of the receiver. So, for example, when the sender sends *M*_1_ in *S*_2_ but the receiver refuses to mate, this is merely a case of attempted deception. If the receiver does mate with the low-quality sender, this is a case of deception.

Existing discussions of cases of this kind routinely assume a concept of deception similar to ours, without spelling out a view of content that licences it. For example, Zollman *et al.* ([2013]) describe the hybrid equilibria that can exist in these signalling games in the following terms: ‘In plain English, this means that the sender sometimes “lies” and is honest at other times, whereas the receiver only sometimes chooses the sender’s favoured action’. If the notion of ‘lying’ requires that a message has false content, and not merely that it withholds some information, then informational content as discussed here and elsewhere does not suffice to make sense of lying, and something like functional content in our sense is needed.

A further notable feature of our treatment of this case is that the functional content of *M*_2_ is undefined, as Table 8 shows. This is because no state of the world generates higher-than baseline payoffs given the receiver's equilibrium response to *M*_2_. Indeed, although *M*_2_ is treated in the model as a signal, it is associated neither with costs nor the possibility of benefit, so it is more naturally understood as the absence of a signal—as a ‘null’ signalling behaviour.

While that is a satisfactory result in the present case, in other games with intrinsic signalling costs, our proposed definition of functional content is more problematic. Bergstrom and Lachmann ([1997]) analyse another game with costly signals, the Sir Philip Sidney game. They show that there are separating equilibria in which both sender and receiver are worse off than they would be if the receiver produces its best cover-all response to completely uninformative signals.^13^ In such a separating equilibrium, there is no state in which both players obtain payoffs above their baseline, as we have defined the baseline; so there is no functional content.

To sketch a response to this problem, we return to the theoretical motivation for our account. Functional content is a matter of more than just coordinating actions with the state of the world. That happens in the case of perfect anti-signalling mentioned above, where signals are perfectly coordinated with world states, but no payoffs result. Functional contents arise where the players coordinate actions with the state of the world successfully. Isolating cases of successful coordination calls for a standard of comparison, which is what our baselines achieve. If one accepts this theoretical motivation, then it follows that functional content is not ubiquitous—it is absent in some equilibria where signals are coordinated with the state of the world.

As formulated, our definition has the consequence that functional content is absent when players fall into an equilibrium in a costly signalling game that makes them worse off in every world state than they would be without signalling (although there is functional content in the costly signalling game we analyse here). Rather than just accepting that consequence, another solution would be to define baselines more locally when there are intrinsic signalling costs, in terms of nearby states in which both sides do worse than at the equilibrium. We do not attempt to resolve this issue here.

### 4.6 Case 6: A further problem arising from divergent interests

When sender and receiver interests diverge, but do not diverge greatly, a problem can arise that has not been addressed in our cases above. This problem comes when, given some act or mix of acts produced in response to a message at equilibrium, sender and receiver both achieve above-baseline payoffs in the same combination of states, but the degrees to which they benefit in each of these states differ. Then when a vector representation of functional content is given, strictly speaking there will be one vector for the sender and one for the receiver, not a single vector describing both. This does not happen in either of the two cases with divergent interests discussed above. In one of these cases (Case 5), no message is interpreted in a way that gives both parties an above-baseline payoff in more than one state (only the sender receives an above-baseline payoff in more than one state, given the receiver’s rule for *M*_1_). In the other Case 4, both sender and receiver obtain above-baseline payoffs in *S*_1_ and *S*_2_, given the rules associated with *M*_1_, and these payoffs do differ between sender and receiver, but for neither agent is one state preferable to the other. So there is no qualitative difference between the agents with respect to the roles of *S*_1_ and *S*_2_ in stabilizing this aspect of their interaction.

In other possible cases, when the interests of the agents diverge in a way that leads to a message being associated with more than one state of the world for each agent, though with different weightings for these states across the two agents, the formulation we give is designed to capture the ‘overlap’ between sender and receiver interests (see the appendix for details). As we noted, in such cases it is also straightforward to record separate functional content vectors for sender and receiver, respectively. Comparisons between our preferred functional content vector, which captures the overlap, and the separate functional content vectors for sender and receiver would show the respects in which sender and receiver have different interests in the way the signal is connected to world states at equilibrium.

**Figure 6.**
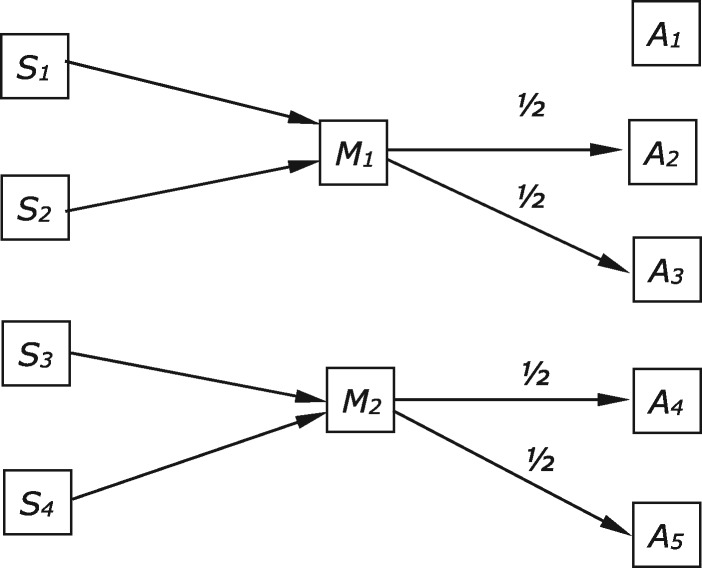
Sender and receiver behaviours in the equilibrium considered in Case 6.

A case put forward by a referee helpfully illustrates another way this kind of divergence can arise. In this new case, there are four equiprobable states, five available acts, and two costless signals, with payoffs as given in Table 9. We focus on the equilibrium shown in Figure 6, in which both players receive an above-baseline payoff in one particular state for each signal, but the relevant state differs for each player. For example, when *M*_1_ is sent, the sender receives a payoff only in *S*_1_ and the receiver only in *S*_2_. Our ‘overlap’ functional content vector is undefined. Table 10 records separate functional content vectors for sender and receiver.

**Table 9.** Payoffs in Case 6

		Acts				
		*A*_1_	*A*_2_	*A*_3_	*A*_4_	*A*_5_
States	***S***_1_	2, 2	5, 0	5, 0	0, 0	0, 0
	***S***_2_	2, 2	0, 5	0, 5	0, 0	0, 0
	***S***_3_	2, 2	0, 0	0, 0	5, 0	5, 0
	***S***_4_	2, 2	0, 0	0, 0	0, 5	0, 5

Payoffs in each cell are to sender and receiver, respectively.

**Table 10.** Relations between informational and functional content for Case 6

		Informational content	Functional content for sender	Functional content for receiver
Messages	*M*_1_	<0.5, 0.5, 0, 0>; *S*_1_*-or-S*_2_	<1, 0, 0, 0>; *S*_1_	<0, 1, 0, 0>; *S*_2_
	*M*_2_	<0, 0, 0.5, 0.5>; *S*_3_*-or-S*_4_	<0, 0, 1, 0>; *S*_3_	<0, 0, 0, 1>; *S*_4_

Contents are given first in vector form and then in a narrative summary.

Comparing the separate functional content vectors for sender and receiver, and in the absence of an ‘overlap’ functional content vector, we can see that the two players have completely different interests in the way the signal is connected with world states at equilibrium. The receiver is only interested in the way *M*_1_ carries information about state *S*_2_, whereas the sender receives a payoff only when *S*_1_ obtains. An alternative perspective on this case would be to argue that sender and receiver do share an interest when *M*_1_ is sent—an interest in the fact that *S*_1_-*or-S*_2_ obtains. A natural move here would be to describe the game in a more coarse-grained way, so that *S*_1_-*or-S*_2_ counts as a single state. Sender and receiver would then overlap in functional content with respect to that state. The difficulty is to formulate a rule for when it is appropriate to move to a more coarse-grained functional content vector that does not have the result that all cases of partial pooling turn into cases of perfect signalling with more coarse-grained states. While this case is clearly another reason to distinguish functional contents for sender and receiver in some cases, we have no settled view as to whether there is also a principled way to define a non-vacuous overlap functional content vector in this case.

## 5 Discussion

The small selection of examples above show that there is an important role for a notion of content that goes beyond purely informational content, even in these simple cases. Specifically, there is a role for a treatment that is connected to equilibria and how they are stabilized by payoffs. The way theorists routinely talk about simple signalling systems makes this clear. They say things that implicitly draw on a richer notion of content than informational content. This might be seen as metaphorical. But we have shown that a concept like this can be made precise and shown to be useful, especially in contexts where false content is important, such as in the analysis of deception.

Teleosemantics also aimed to capture the involvement signs have with the world. The concept of functional content developed here is a fine-grained take on that idea. The need to go beyond a purely informational treatment and introduce a broadly functional notion of content is one of the insights of Millikan ([1984]), Papineau ([1993]), and Dretske ([1988]). What we're doing is combining those ideas with Skyrms's introduction of a fine-grained vector representation of content. Our functional content vector captures the relative importance of different states when more than one state is involved in stabilizing a pattern of sender and receiver behaviours.

The concept of functional content we have developed here is not the only way this could be done. And it is clear that our treatment in this article still faces some problems. We hope to have shown that it is widely applicable enough to illustrate that there is space for an account of functional content alongside that of informational content.

Lastly, we make a comment about the status of these properties, which we have been calling a kind of ‘content’. Clearly the signs themselves and their associated behaviours are much simpler and more rudimentary than those associated with human language and thought. They are probably simpler than most non-human sign systems as well. We don't claim that informational and functional content exhaust the rich semantic properties seen in language and thought. They can be thought of as simpler members of a family of semantic properties, or as precursors to real semantic properties. These simpler semantic or proto-semantic properties are, however, important features of signalling systems. Our notion of functional content captures a theoretically important aspect of sender–receiver interaction.

## Footnotes

^1^ (Lewis [1969]; Spence [1973]; Crawford and Sobel [1982]; Robson [1990]; Farrell and Rabin [1996]; Bergstrom and Lachmann [1998]; Maynard Smith and Harper [2003]; Huttegger *et al.* [2010]; Skyrms [2010]; Clark [2011]; Stegmann [2013]; Zollman *et al.* [2013]).

^2^ Huttegger’s ([2007b]) work on the distinction between indicative and imperative content also suggests that there is a role for functional considerations in defining content; see also (Zollman [2011]). These discussions may lead in the direction of an alternative notion of functional content to the one presented in the present article.

^3^ Indeed, Skyrms ([2010], p. 47) himself sometimes privileges the kind of information flow found at equilibrium—where the receiver ‘acts just as she would have if she had observed the state directly’ over other cases where just as much information is transmitted.

^4^ In the literature on ‘functional reference’ in animal communication, mentioned above, Scarantino ([2013], p. 1016) does the same in combining a ‘contextual perception criterion’ (dependent on evolutionary functions) with a ‘contextual information criterion’.

^5^ Harms ([2004]) was perhaps the first to connect sender–receiver models with a functional notion of content.

^6^ Birch ([2014]) uses a different way of defining content to argue that signals in out-of-equilibrium states have propositional content (which, as with our functional content, in general differs from informational content).

^7^ This is equivalent to weighting the posterior probabilities by a function of the payoffs.

^8^ (Barrett [2006]) is the first discussion of pooling equilibria for signalling games such that, for any number, *n*, there are *n* states, *n* signals, and *n* acts; see also (Barrett [2007]).

^9^ In the cases we are considering, *x* and *y* are non-zero.

^10^ For a discussion of measures of the degree of common interest based on divergence between the sender's and receiver's preference orderings over actions in states, see (Godfrey-Smith and Martínez [2013]).

^11^ In the previous payoff matrix (Table 3), there was one entry for both sender and receiver payoffs in a combination of act and state. In Table 5, each cell contains a pair of numbers, for sender and receiver payoffs, respectively, for the corresponding act and state.

^12^ The equilibrium requires that the probability of the receiver accepting the high-cost signal (that is, performing *A*_1_ in response to *M*_1_) is equal to the cost to low-quality senders of sending the high-cost signal (that is, of sending *M*_1_ in *S*_2_). Both are equal to ½ in our illustration. This has the effect of ensuring that the benefit to low-quality individuals of sometimes achieving a mating is exactly balanced, on average, by the cost to low-quality individuals of sending the high-cost signal.

^13^ We are grateful to a referee for pointing out the implications of this game.

## Appendix

Here we will provide the definition of the functional content vector.

$$Functional\;content\;of\;signal\;M=\;<\frac{x_{1}}{\sigma },\;\frac{x_{2}}{\sigma },\;\ldots ,\;\frac{x_{n}}{\sigma }>.$$

We define the functional content vector for an arbitrary signal *M*, following the procedure given in Section 3.2 above. The vectors listed in the case studies above are the result of applying this procedure to each signal *M*_1_, *M*_2_, … found in the model. Below we proceed in two parts. First we define the baseline payoff for a signalling game. The baseline is then used as a threshold to generate components of the functional content vector.

### A.1 Baseline Payoffs

We define the functional content vector in relation to the baseline payoffs obtained for the sender and receiver in the absence of signalling. Baseline for each (${\bar{v}}_{r}$, ${\;\bar{v}}_{s}$) is its expected payoff given *A**, the action dictated by the best strategy the receiver can adopt without conditioning its behaviour on any signals.^14^ In defining the baseline here, we consider only pure receiver strategies since in the absence of signals, the receiver can never do better by mixing than by pursuing some pure strategy.

$$\mathrm{Receiver's}\;\mathrm{baseline}\;\mathrm{payoff},{\bar{v}}_{r}=\sum_{i=1}^{n}P\left(S_{i}\right)v_{r}\left(A^{*}|{|S}_{i}\right),$$

$$\mathrm{Sender's}\;\mathrm{baseline}\;\mathrm{payoff},{\bar{v}}_{s}=\sum_{i=1}^{n}P\left(S_{i}\right)v_{s}\left(A^{*}||S_{i}\right),$$

$$v_{r}\left(A^{*}||S_{i}\right)=\mathrm{Receiver's}\;\mathrm{payoff}\;\mathrm{for}\;\mathrm{action}\;A^{*}\;\mathrm{when}\;\mathrm{world}\;\mathrm{is}\;\mathrm{in}\;\mathrm{state}\;S_{i},$$

$$v_{s}\left(A^{*}||S_{i}\right)=\mathrm{Sender's}\;\mathrm{payoff}\;\mathrm{for}\;\mathrm{action}\;A^{*}\;when\;world\;is\;in\;state\;S_{i},$$

$$n=number\;of\;world\;states\;S_{i}.$$

### A.2 Functional Content Vector

Components ${\;x}_{i}$ in the functional content vector reflect the average payoff received from world state $S_{i}$ when signal *M* is sent, thresholded by reference to the baseline payoffs calculated above. Non-zero entries correspond to states in which both agents receive above-baseline payoffs given the receiver’s rule for *M*, and record the amount by which the threshold is exceeded. The requirement that both agents receive above-baseline payoffs implies that the agents have similar payoff matrices to some degree, but differences are still possible. Accordingly, we construct the vector entries by using the lesser of the amounts by which the two agents’ payoffs surpass their baseline: $d_{min}\left(A_{j}||S_{i}\right)$. This is designed to represent the overlap between the sender’s and receiver’s interests:

$$d_{min}\left(A_{j}||S_{i}\right)=\underset{}{\min}\left({(v}_{r}\left(A_{j}||S_{i}\right)-{\bar{v}}_{r}),{(v}_{s}\left(A_{j}||S_{i}\right)-{\bar{v}}_{s})\right).$$

This formulation is appropriate when there is a single population of agents that play the sender role half the time and the receiver role half the time. If there are separate populations of senders and receivers, then the payoffs in the payoff matrix should be transformed to a common scale. Before calculating the baselines and $d_{min}$, the sender's payoffs should be linearly transformed so that its maximum payoff is one, and the same for the receiver. For simplicity we do not include such transformations in the definition below.

The entries in the functional content vector are then defined as follows:

 xi={PSiM{∑j=1mPAj|MdminAjSi},   if ∑j=1mPAj|MvrAjSi>v¯rand∑j=1mPAj|MvsAjSi>v¯s 0,otherwise 

*m = *number of available actions *A_j_*

Finally, the components are normalized by σ so that, as with the informational content vector, they sum to one:

$$\sigma =\sum_{i=1}^{n}x_{i}.$$

The use of $d_{min}$ terms is only essential in special cases; see Section 4.6 of the main text for discussion. In such cases, another option would be to define separate functional content vectors for sender and receiver when they differ. We do not hold that one of these approaches is better than the other; each might represent different features of these cases.
